# Commentary: Do motivations for using Facebook moderate the association between Facebook use and psychological well-being?

**DOI:** 10.3389/fpsyg.2015.01483

**Published:** 2015-09-30

**Authors:** Sherry H. Stewart

**Affiliations:** Psychology and Neuroscience, Dalhousie UniversityHalifax, NS, Canada

**Keywords:** Facebook use, moderation, mediation, addiction, psychological well-being, motives, coping motives

Rae and Lonborg's (2015) findings are intriguing. They show greater Facebook (FB) use intensity can have beneficial or adverse effects on psychological well-being (PWB) depending on the user's motives. This underlines that not all motives are equal: some motives are harmful and others helpful when it comes to PWB and psychopathology outcomes. Four central issues were raised: (1) exclusive focus on social (external) motives for FB use; (2) exclusive focus on PWB outcomes; (3) interesting pattern of findings in the supplemental analyses; and (4) exclusive focus on FB use motives as moderators. Each suggests exciting future possibilities for this nascent area of motives research.

## Exclusive focus on social motives for FB use

Rae and Lonborg's finding that friendship motives had moderating effects in improving PWB among heavier FB users while connection motives had moderating effects in worsening PWB among heavier FB users illustrates that some social motives for use can be beneficial and others harmful. Alcohol research has repeatedly shown that while social-affiliative drinking motives are associated with lighter, non-problematic alcohol use, social-conformity motives are associated with more problematic alcohol use (Cooper et al., 2015). In Cooper's (1994) motivational model of alcohol use, social-affiliative motives (drinking to affiliate with others) involve drinking to achieve an external goal and positive reinforcement; these motives seem to have their parallel in friendship motives for FB use where FB use serves the goal of affiliating with existing friends. Cooper's social-conformity drinking motives (drinking to reduce social censure) also involve drinking to achieve an external goal, but involve negative reinforcement. Social-conformity drinking motives seem to have their parallel in connection motives for FB use where individuals use FB to make new friendships in order to reduce loneliness and isolation.

However, Rae and Lonborg only examined external/social motives for FB use. Cooper's model includes positive and negative reinforcement motives which are internal in nature—where the behavioral goal is to change one's internal state, whether it be increasing positive emotions (enhancement drinking motives) or decreasing negative emotions (coping or escape motives). One can imagine these two drinking motives having parallels in reasons for using FB. Moreover, such internal motives for FB use might be associated with elevations in psychopathological outcomes as has been shown in the substance abuse area (Cooper et al., 2015). Recent research has shown that FB use motivated by escape reasons is associated with greater risk for FB addiction (Masur et al., 2014). I encourage cross-fertilization of ideas across the addictive behaviors and social media literatures when it comes to motivational models/measures; much can be learned from work in the other field.

## Exclusive focus on PWB outcomes

While Rae and Lonborg's focus was on the moderating effects of different FB use motives on the association between FB use and PWB outcomes, including depression and anxiety, other psychopathological outcomes could be investigated in a similar manner. Take for example FB “addiction” (Griffiths et al., 2014). It is possible that the association between FB involvement and FB addiction could be moderated by reasons for FB use. Heavier FB use might be more likely to result in FB addiction if one is using FB for maladaptive motives (e.g., escape motives, connection motives) vs. using FB for more adaptive motives (e.g., friendship). While the Rae and Lonborg paper was designed to shed light on PWB outcomes, a focus on other outcomes would be an intriguing direction for future research.

## Interesting patterns in the supplemental analyses of individual PWB domains

Rae and Lonborg's supplementary analyses at the level of individual PWB scales revealed some noteworthy patterns. First, while friendship motives mainly operated as moderators of FB intensity effects on *positive* aspects of PWB (life satisfaction), connection motives mainly operated as moderators for *negative* aspects of PWB (e.g., anxiety). This suggests that positive reinforcement social motives may exert their moderating influences mainly in *enhancing positive aspects of PWB* whereas negative reinforcement social motives may exert their moderating influences mainly in *intensifying negative aspects of PWB*. Second, while friendship motives tended to interact with *number of friends* in predicting PWB outcomes, connection motives tended to interact with *time spent using FB* in predicting PWB outcomes. This suggests that positive and negative reinforcement social motives for FB use may exert their moderating effects on different aspects of FB use intensity in impacting PWB. These possibilities could be investigated more rigorously using structural equation modeling where all variables are entered into a single model.

## Moderation vs. mediation

While the Rae and Lonborg paper examined moderation, it is also possible to consider the potential role of FB use motives as mediator variables. In Cooper's (1994) motivational model of addictive behaviors (which has been expanded recently from alcohol to other drugs and to gambling; Stewart and Zack, 2008; Cooper et al., 2015), motives are seen as the final common pathway to addictive behavior outcomes through which the effects of other distal risks are mediated. This model may also apply to FB addiction, for example. Roberts and Pirog (2013) showed impulsivity is a predictor of technological addiction propensity in young adults. It is possible that impulsivity is related to risk for FB addiction by way of its association with certain maladaptive motivations for using FB. In the alcohol field, impulsivity's association with risk for problematic drinking is mediated through impulsive individuals' tendency to drink for enhancement motives (Mackinnon et al., 2014). A similar mediational model could be tested for impulsivity, FB motives, and FB addiction. Recent work supports the mediating role of specific motives for FB use when explaining the link of certain individual differences and FB addiction. Masur et al. (2014) found escape motives mediated the relation of lack of autonomy to FB addiction whereas connection motives mediated the association between lack of relatedness and FB addiction.

Additional work could be conducted on other potential moderator models (e.g., moderating effect of PWB on the relationship between motives and FB addiction). Kardefelt-Winther (2014) showed PWB moderated the effect of escapism motives for online gaming to gaming-related negative outcomes. Specifically, the relationship between escapism and negative outcomes was positive only for low PWB levels among those with more negative outcomes. PWB may have a similar moderating effect on the relation between connection motives and excessive FB use.

Both moderator and mediator findings are important in directing intervention work. Moderator studies identify *whom* to focus on for intervention delivery (i.e., high intensity FB users who use primarily for connection purposes). Mediator studies identify *what* to focus on (e.g., reducing connection motives for FB use and building healthier ways for FB users, lacking in relatedness, to form new friendships).

### Conflict of interest statement

The author declares that the research was conducted in the absence of any commercial or financial relationships that could be construed as a potential conflict of interest.
